# Gr1^int^CD11b^+^ Myeloid-Derived Suppressor Cells in *Mycobacterium tuberculosis* Infection

**DOI:** 10.1371/journal.pone.0080669

**Published:** 2013-11-01

**Authors:** Andrés Obregón-Henao, Marcela Henao-Tamayo, Ian M. Orme, Diane J. Ordway

**Affiliations:** Mycobacteria Research Laboratories, Department of Microbiology, Immunology and Pathology, Colorado State University, Fort Collins, Colorado, United States of America; University of Pittsburgh, United States of America

## Abstract

**Background:**

Tuberculosis is one of the world’s leading killers, stealing 1.4 million lives and causing 8.7 million new and relapsed infections in 2011. The only vaccine against tuberculosis is BCG which demonstrates variable efficacy in adults worldwide. Human infection with *Mycobacterium tuberculosis* results in the influx of inflammatory cells to the lung in an attempt to wall off bacilli by forming a granuloma. Gr1^int^CD11b^+^ cells are called myeloid-derived suppressor cells (MDSC) and play a major role in regulation of inflammation in many pathological conditions. Although MDSC have been described primarily in cancer their function in tuberculosis remains unknown. During *M. tuberculosis* infection it is crucial to understand the function of cells involved in the regulation of inflammation during granuloma formation. Understanding their relative impact on the bacilli and other cellular phenotypes is necessary for future vaccine and drug design.

**Methodology/Principal Findings:**

We compared the bacterial burden, lung pathology and Gr1^int^CD11b^+^ myeloid-derived suppressor cell immune responses in *M. tuberculosis* infected NOS2-/-, RAG-/-, C3HeB/FeJ and C57/BL6 mice. Gr-1^+^ cells could be found on the edges of necrotic lung lesions in NOS2-/-, RAG-/-, and C3HeB/FeJ, but were absent in wild-type mice. Both populations of Gr1^+^CD11b^+^ cells expressed high levels of arginase-1, and IL-17, additional markers of myeloid derived suppressor cells. We then sorted the Gr1^hi^ and Gr1^int^ populations from *M. tuberculosis* infected NOS-/- mice and placed the sorted both Gr1^int^ populations at different ratios with naïve or *M. tuberculosis* infected splenocytes and evaluated their ability to induce activation and proliferation of CD4+T cells. Our results showed that both Gr1^hi^ and Gr1^int^ cells were able to induce activation and proliferation of CD4+ T cells. However this response was reduced as the ratio of CD4^+^ T to Gr1^+^ cells increased. Our results illustrate a yet unrecognized interplay between Gr1^+^ cells and CD4^+^ T cells in tuberculosis.

## Introduction

 Tuberculosis is the primary cause of death from a bacterial disease, and is further exacerbated by the very extensive incidence of latent disease, as well as the emergence of drug-resistant forms of the bacillus [1,2]. Animal models have provided much information regarding the pathogenesis and host response to the disease process [3], but a limitation of the most widely used model, the mouse, is the lack of lung necrosis [4], the hallmark of human tuberculosis. Necrosis is the central and eventually fatal event in the pathogenesis of the disease [5-7] and provides a safe niche in which bacilli surviving the initial wave of acquired immunity can persist [5,8,9]. If, as in humans and in some cases guinea pigs [10] the lesion cavitates, further transmission of the disease can ensue.

 That is not to say however that some more recently described inbred mouse models do not develop necrosis. Chronically infected mice on the C3Heb/FeJ background, for instance, gradually develop degenerating lesions. Mice in which genes have been deleted for gamma interferon, B and T cells [11], GM-CSF [12], and NOS2 [13] all develop severe lung necrosis after low dose aerosol infection. More recently, it has been shown that C3HeB/FeJ and I/St murine strains undergo pulmonary lung necrosis during *M. tuberculosis* infection [14,15]. 

 There is increasing evidence that Gr1+ neutrophils are present in the granuloma and play a key role in the process of necrosis[5]. They are among the first cells to enter lesions [16,17], where they degranulate to form microfoci of eosinophilic debris which we suspect coalesce to form the central necrosis in the characteristic granuloma structure. They also produce reactive oxygen radicals which have little effect on the bacilli but can damage the integrity of the local vasculature and surrounding tissue [18]. Their influx drops as acquired immunity expands [19] but if this wanes again, as can be observed in the guinea pig during chronic infection [20], they arrive anew. In human tuberculosis, they are the predominant population found in the airways and contribute to airway transmission [21]. Furthermore, markers associated with neutrophils predominate in a transcriptional analysis of blood from tuberculosis patients [22]. However, we have yet to fully understand neutrophils role in *M. tuberculosis* disease. 

 In the studies reported here, we used flow cytometry and cell sorting techniques to monitor the influx of granulocytes into the lungs of necrosis prone mouse strains NOS2-/-, RAG-/-, C3HeB/FeJ strains and wildtype C57BL/6 mice devoid of necrosis. We tracked the influx of Gr1+ cells to the lungs and demonstrate two distinct populations: one Gr-1^hi^, and a second, quite substantial Gr-1^int^ population. Gr-1^int^ cells have been described primarily in cancer, and are thought to be a more immature cell type, but there is evidence they can modulate both innate and acquired responses [23-25].These cells are a heterogeneous group of myeloid cells that are capable of regulation of inflammation in pathological conditions [26]. Ample evidence exists that MDSC have a reciprocal relationship with CD4+ T cells being able to induce activation and proliferation or suppression [26] Our studies demonstrate Gr^high^ and Gr1^int^ cells from necrosis-prone mice have properties of MDSCs [27], such as expression of Ly6c, CD14, F4/80, arginase I and IL-17. Furthermore, MDSCs from infected mice are capable of inducing activation and proliferation of CD4+ T cell cells and well as inhibiting proliferation showing a yet unidentified role for these cells in tuberculosis. 

## Results

### Evidence for an increase in CD11b+ but not CD11c+ cells in infected NOS2-/- mice

**Figure 1:**
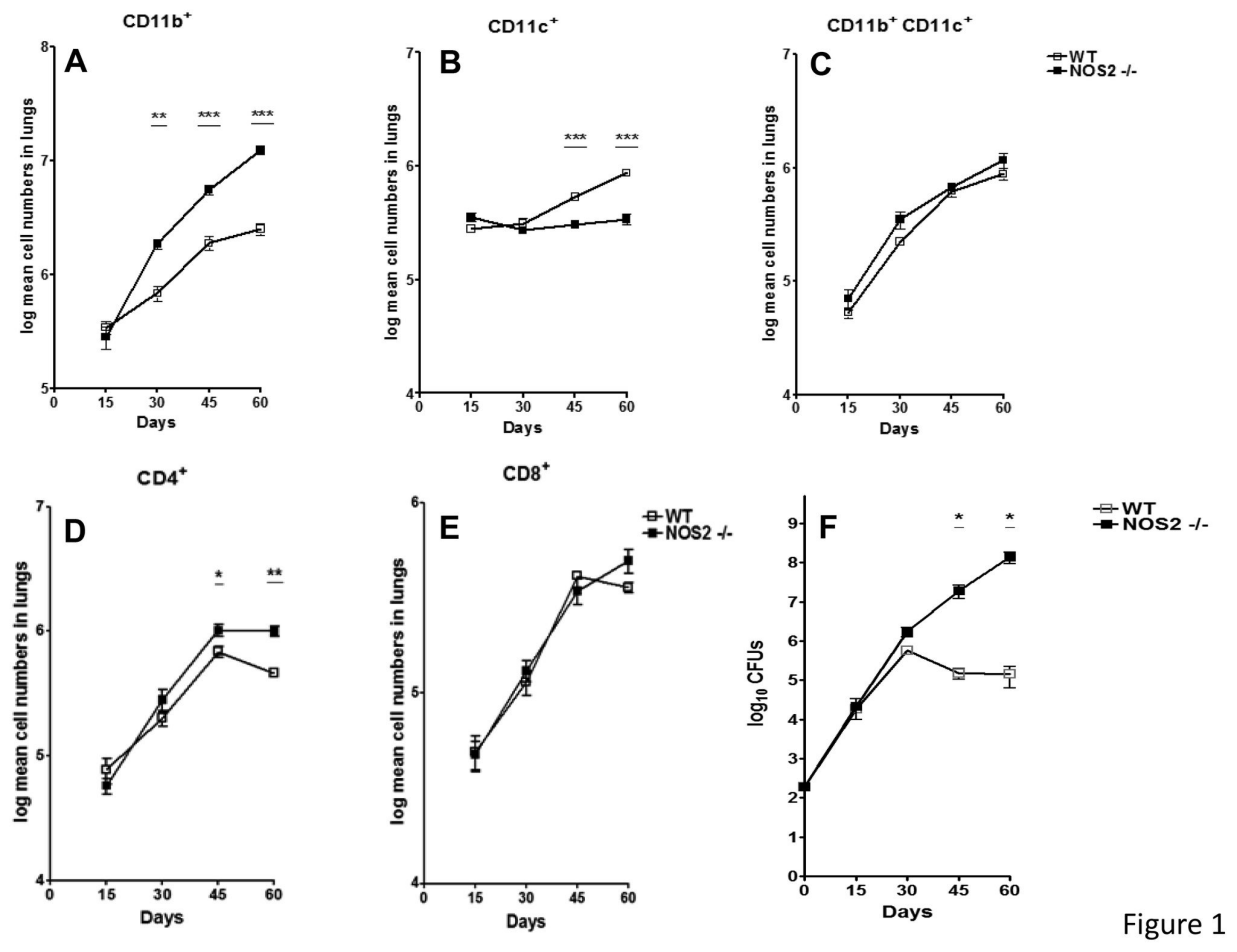
Higher numbers of CD11b^+^ cells in the lungs of NOS2-/- KO mice. Flow cytometry analysis was performed on single cell suspensions obtained from lungs of WT C57/BL6 or NOS2-/- mice. In these mice increased numbers of CD11b^+^ cells but not CD11b^+^CD11c^+^ cells could be observed as the infection progressed (A, B, C). In contrast, differences for CD11c^+^ and CD4^+^ cells only became evident at later time points (B, D). No significant differences were observed for CD8^+^ cells (E). Results are expressed as the mean values of log mean cell number (± SEM, n=5) in the lung. Bacterial burden (F) was enumerated at different time points after a low dose aerosol infection with *M. tuberculosis*. As observed, bacterial burden in both murine strains is similar at early time points after infection and diverges after 30 days of infection. Results are expressed as the mean Log 10 CFUs (±SEM, n=5) in the lungs. Student t-test, * p<0.05, ** p<0.01, *** p<0.001.

Flow cytometric analysis was performed on gene disrupted and wild-type (WT) mice in order to analyze the influx of leukocytes into the lungs. From day 30 onwards, a time at which studies have previously demonstrated [13] that the gene disrupted mice start to fail to contain the infection (Figure 1, F), we observed an accelerated increase in cells staining positive for CD11b+ but no significant differences in cells staining for CD11b+ CD11c+ (Figure 1A, C). This was consistent with the presence of granulocytes, as previously described [28]. Furthermore, the numbers of CD11c+ and CD4+ T cells (Figure 1B, D) were only affected at later time points during chronic infection, whereas no difference was observed through the course of infection for CD8+ T cells (Figure 1E).

### Characterization of expression of Gr-1 delineates two major populations

**Figure 2:**
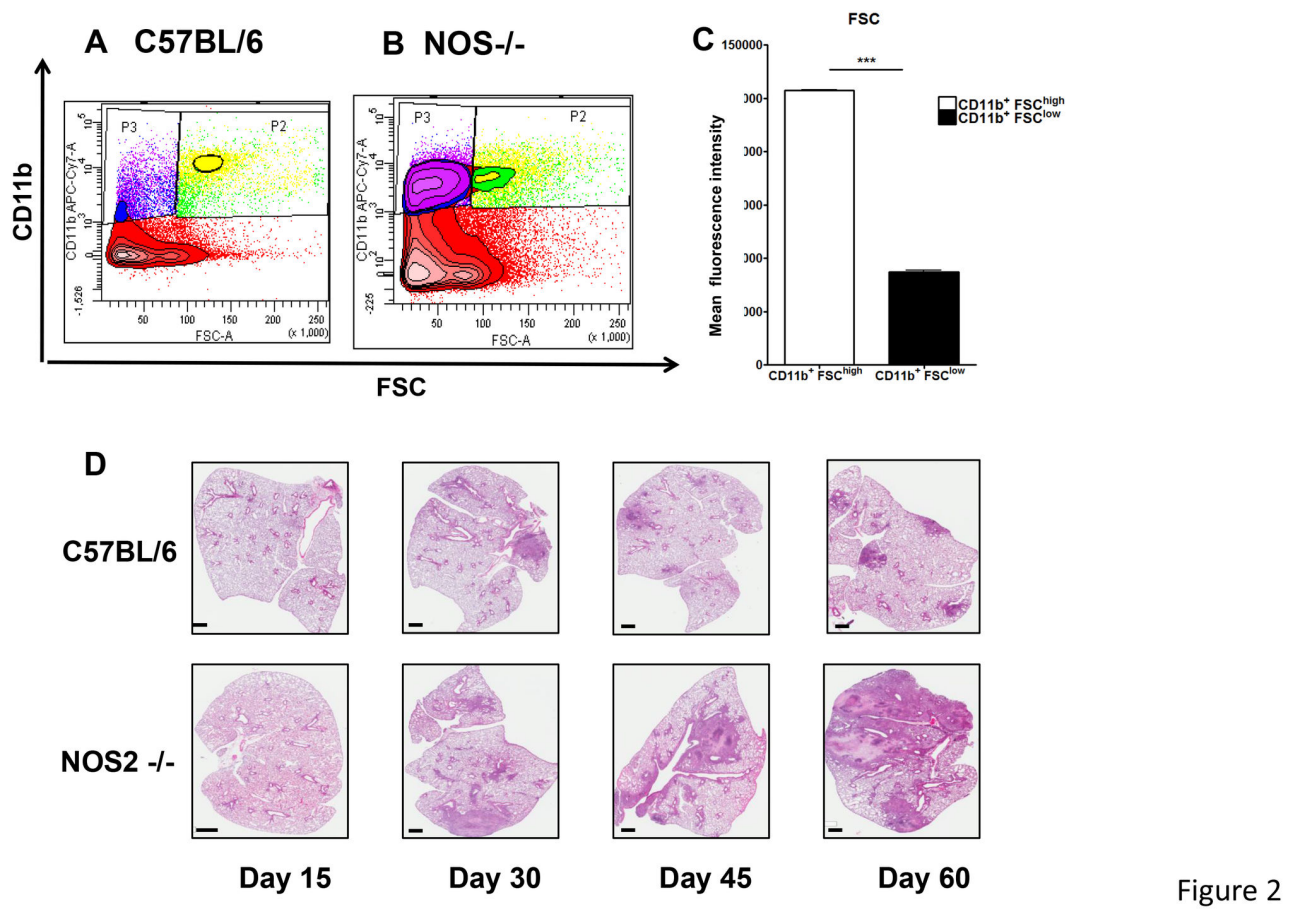
Two populations of CD11b^+^ cells based on FSC analysis. As suggested by FSC analysis, the increased number of CD11b^+^ cells present in NOS2 -/- mice could be separated in two populations based on cell size (A, B). Both populations of CD11b^+^ FSC^high^ cells and CD11b^+^FSC^low^ cells are significantly increased in NOS2 -/- mice compared to C57/BL/6 mice (A, B). Contour plot is representative of day 45 after infection. FSC analysis clearly identified two CD11b^+^ populations in the lungs of NOS2 -/- mice (C). Results are expressed as the mean values fluorescence intensity (± SEM, n=5) in the Lung, Student t-test, *** p<0.001. Necrotic granulomas are only present in the lungs of NOS2 -/- mice (D). At different time points after infection, lungs were harvested and stained with H&E after fixation and paraffin embedding. For both murine strains, lesions are not present at day 15 after infection. Starting day 30, necrotic granulomas become evident in the lungs of NOS2 -/- mice but not WT C57BL/6. As observed at day 45 and 60, necrotic granulomas coalesce leading to severe lung consolidation at day 60 when most NOS2 -/- are moribund.

**Figure 3:**
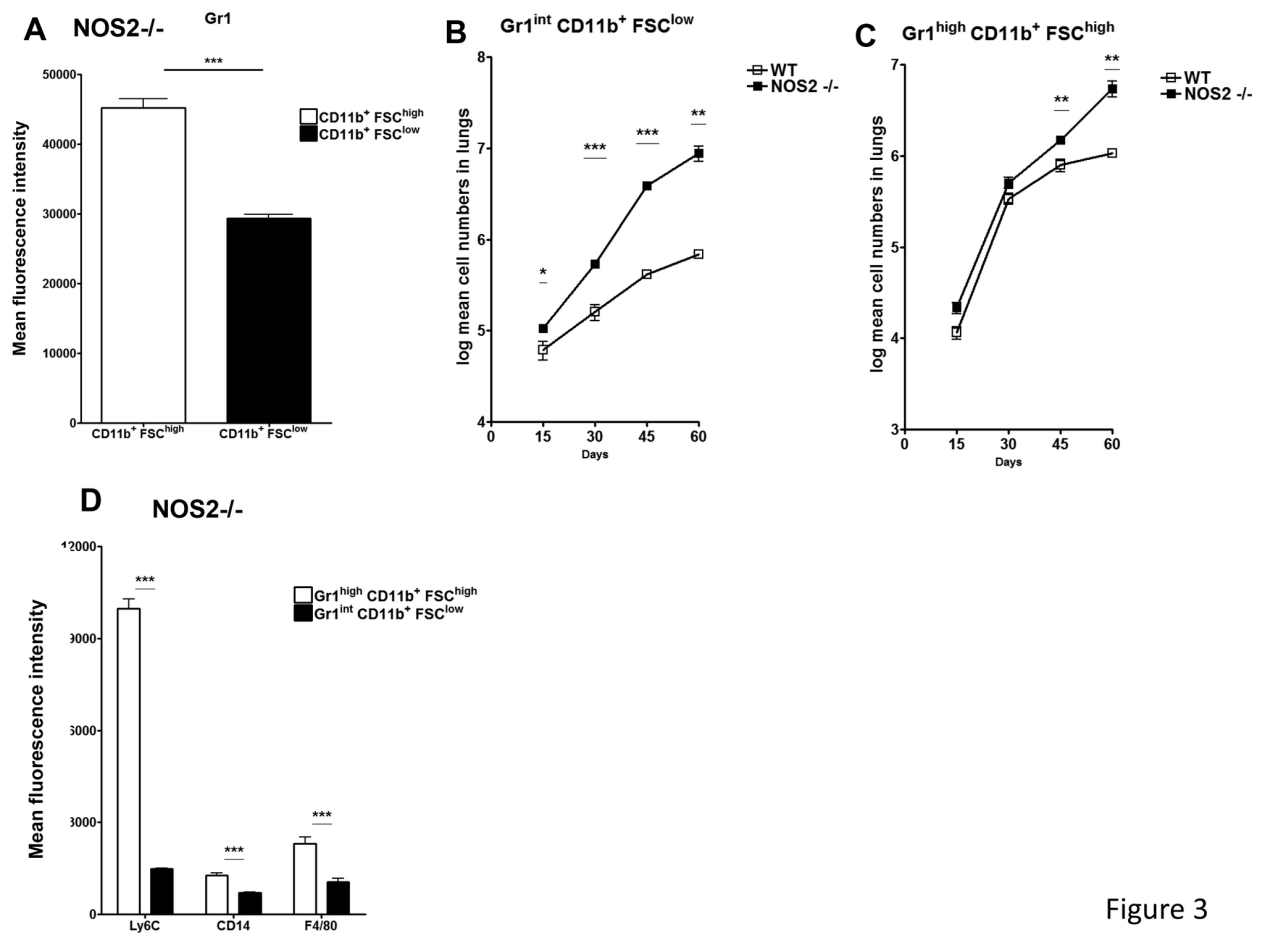
Gr1^int^ CD11b^+^ FSC^low^ cells predominate in the lungs of NOS2-/- mice. Further analysis of CD11b^+^FSC^low^ and CD11b^+^FSC^high^ cells was performed by staining with anti-Gr1 antibody. As determined by mean fluorescence intensity, CD11b^+^FSC^high^ had significantly higher Gr1 expression than CD11b^+^FSC^low^ (A). Starting at early time points NOS2-/- mice but not WT C57BL/6 mice have a significant number of cells giving an intermediate staining pattern for Gr-1 (B). At later time points, the influx of CD11b^+^FSC^high^ cells into the lungs of NOS2 -/- mice is also enhanced (C). Results are expressed as the Log mean cell number (± SEM, n=5) in the lung. Gr1^high^ CD11b^+^ FSC^high^ cells express markers compatible with a monocytic lineage. Both populations of Gr1 expressing cells were further analyzed by flow cytometry using the monocytic markers Ly6C, CD14 and F4/80 (D). As determined by the mean fluorescence intensity for these markers, a more precise phenotype for these two cellular populations would be Gr1^high^ CD11b^+^ FSC^high^ Ly6C^high^ CD14^+^ F4/80^+^ and Gr1^int^ CD11b^+^ FSC^low^ Ly6C^low^ CD14^low^ F4/80^low^, compatible with a monocytic and granulocytic lineage, respectively. Results are expressed as the log mean cell number (± SEM, n=5) in the Lung. ***Student t test, * p<0.05, ** p<0.01, *** p<0.001.

In contrast to C57BL/6, flow cytometric analysis clearly identified two CD11b^+^ populations in the lungs of *M. tuberculosis* infected NOS2 -/- mice after 30 days of infection (Figure 2A, B). The populations of GD11b^high^ and CD11b^low^ cells significantly differed in mean fluorescence intensity (MFI) suggested by the difference in CD11b+ verses FSC (Figure 2C). Interestingly, these two CD11b^+^ populations also differed in the degree of Gr1 expression: high Gr1 MFI was observed for CD11b^+^ FSC^high^, whereas CD11b^+^ FSC^low^ cells had intermediate Gr1 MFI (Figure 3 A, B). The increased expression of Gr-1^int^ CD11b^+^ FSC^low^ population in the NOS2-/- mice was associated with the increase in lung pathology present during chronic disease on days 45 and 60 (Figure 2D).

### Increase of Gr-1^int^ CD11b^+^ FSC^low^ population during chronic disease in NOS2-/- mice

As shown in Figure 3, a dominant population of Gr-1^+^ cells accumulating in the lungs of NOS2 -/- mice was identified as Gr1^high^ CD11b^+^ FSC^high^ (Figure 3A). However, when NOS2-/- mice were examined the Gr-1 ^int^CD11b ^+^FSC ^low^ population was increased compared to the C57/BL 6 controls (Figure 3 B). In addition, NOS2-/- mice demonstrated a log mean increase in both Gr1^high^ CD11b^+^ FSC^high^ and Gr-1^int^ CD11b^+^ FSC^low^ cells compared to wild-type C57/BL mice (Figure 3B, C). Based on the high or low expression levels of the Gr1 isoform Ly6-C, MDSCs are commonly divided into monocytic or granulocytic MDSCs respectively. Upon further phenotypic analysis of the two Gr1 populations present in NOS2 -/- mice, Gr1^high^ CD11b^+^ FSC^high^ cells were shown to express high Ly6-C levels (Figure 3D). In contrast Gr-1^int^ CD11b^+^ FSC^low^ cells were Ly6-C low or dim (Figure 3D). Additional markers such as CD14 and F4/80 were also expressed at higher levels in Gr1^high^ Ly6-C^high^ CD11b^+^ FSC^high^ cells, further suggesting a monocytic origin to these MDSCs (Figure 3D).

### Evidence for two distinct morphologically different Gr1^+^ roles

**Figure 4:**
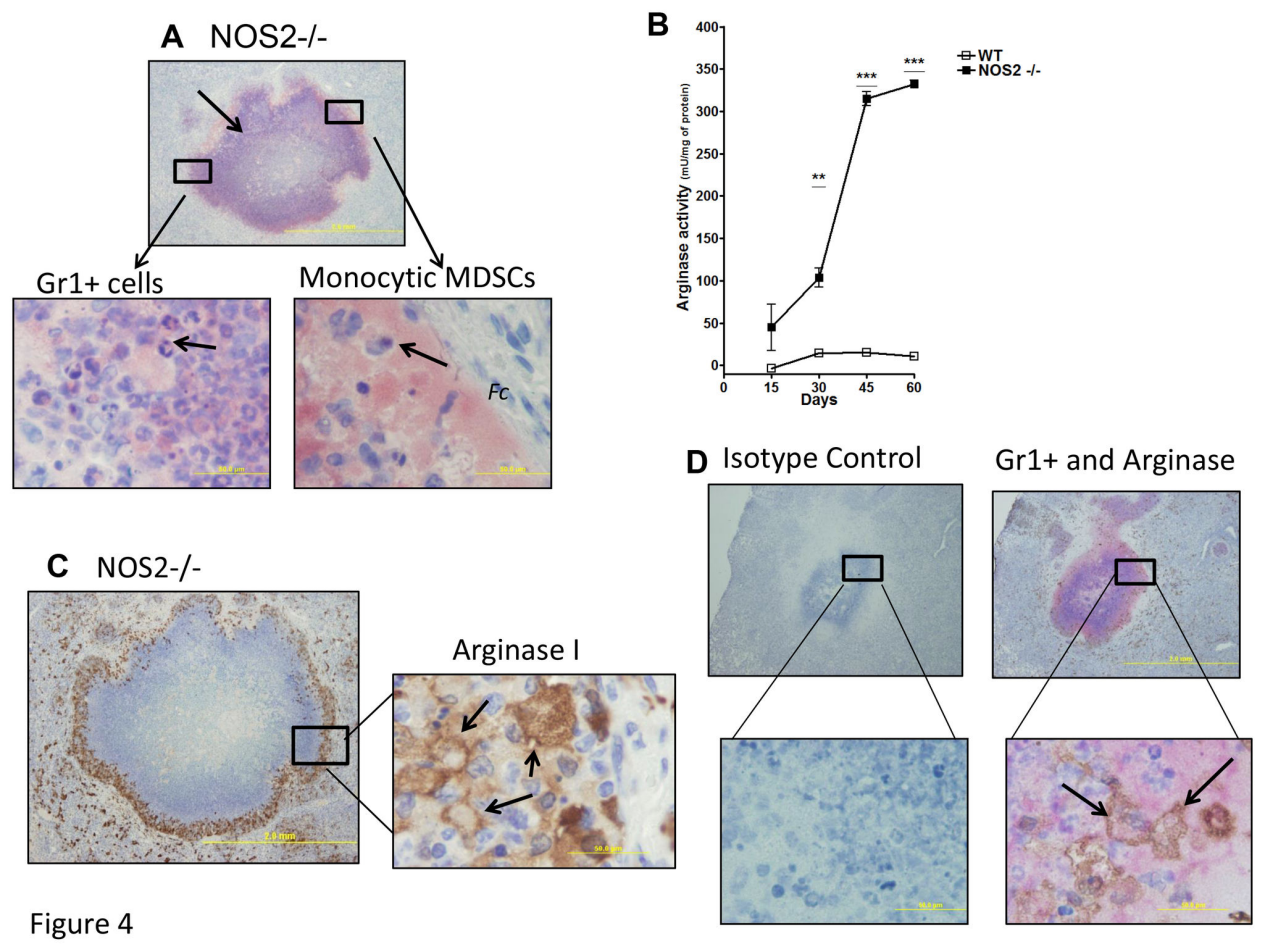
Peripheral localization and distinct morphology of Gr1^+^ cells present in necrotic lungs. Anti-Gr1 immunohistochemistry was performed to determine the location of Gr1^+^ cells surrounding necrotic lesions in the NOS2 -/- mice. As observed in low magnification (4x), anti-Gr1 labeling was predominantly localized in the periphery or rim of these structures (red, arrows, A). At higher magnification (100x), two distinct cellular morphologies could be observed for cells staining with anti-Gr1. In the left panel, neutrophils with multi-lobed nucleus could readily be seen. However, particularly in close apposition to the fibrotic capsule, cells were seen that had abundant and vacuolated cytoplasms as well as a unilobed nucleus, consistent with the morphology of MDSCs. Arginase activity was biochemically detected in the lungs of NOS2 -/- mice but minimally WT controls (B). As disease progressed in NOS2 -/-, more arginase activity could be detected compared to wild-type mice (B).Results are expressed as the mean values of arginase activity (± SEM, n=5) in the Lung. **Student t-test, p<0.01 and ***Student t-test, p<0.001. Anti-arginase-1 immunohistochemistry was performed to determine its expression by inflammatory cells in the lungs of NOS2-/- mice. Similar to the anti-Gr1 staining, at low magnification (4x), robust arginase-1 expression was detected in cells located in the rim of necrotic granulomas (C, left panel). At higher magnification (100x), arginase-1 expression was limited to highly vacuolated cells containing abundant cytoplasm (C, right panel). Panel D shows co-localized staining of both Gr1+ and arginase-1 in NOS2 -/- mouse lung tissues after 45 days of infection. At higher magnification (100x), co-localized staining of Gr1+ and arginase-1 demonstrates that Gr1+ cells (D lower right panel, arrow, red) and arginase-1 staining (D, arrow, brown) are clearly co-localized on the same cell.

 To evaluate the kinetics of migration, localization and morphology of Gr1^+^ cells in the lungs of infected NOS2 -/- mice, immunohistochemical analysis was performed in tissues 45 days after infection. As shown in Figure 4, reactivity with anti-Gr1 was predominantly localized to the rim of necrotic granulomas (Figure 4A, red, arrow). Corroborating the flow data, two distinct cellular populations could be observed to stain with the anti-Gr1 antibody at higher magnifications (Figure 4A). As expected, neutrophils with multi-lobed nucleus were easily identified infiltrating the necrotic granulomas (Figure 4A, arrow, left panel). However, in addition large numbers of Gr1+ cells were also observed in close proximity to the fibrous capsule (Fc) (Figure 4A, arrow, *Fc*), forming a clear boundary between both regions. In contrast to neutrophils, the second population of cells resembled monocytes with a unilobed nucleus and a very extensive vacuolated cytoplasm. This morphology is very similar to other reports describing monocytic MDSCs in tumors or hypoxic conditions [29]. 

 Arginase-1 activity plays a pivotal role in MDSCs function [27]. Thus, the expression of arginase activity was evaluated biochemically and through immunohistochemistry in the lungs of NOS2 -/- and WT C57/BL6 mice. In contrast to WT C57/BL6 mice, arginase activity in the lungs of NOS2 -/- mice increased linearly with disease progression (Figure 4B). At later time points during chronic disease, arginase activity in NOS2 -/- mice was almost 10-fold higher levels than WT (Figure 4B). Consistent with this result, arginase-1 expression was detected at higher levels *in situ* in the lungs of NOS2 -/- mice (Figure 4C). Arginase-1 expression was predominantly localized to the rim surrounding necrotic granuloma, specifically in large vacuolated cells with abundant cytoplasm. Figure 4D shows co-localized staining of both Gr1+ and arginase-1 in NOS2 -/- mouse lung tissues after 45 days of infection. Co-localized staining of Gr1+ and arginase-1 demonstrates that Gr1+ cells (Figure 4D lower right panel, arrow, red) and arginase-1 staining (Figure 4D, arrow, brown) are clearly co-localized on the same cell. 

### Gr1^int^ cells are increased in other murine models showing lung necrosis

**Figure 5:**
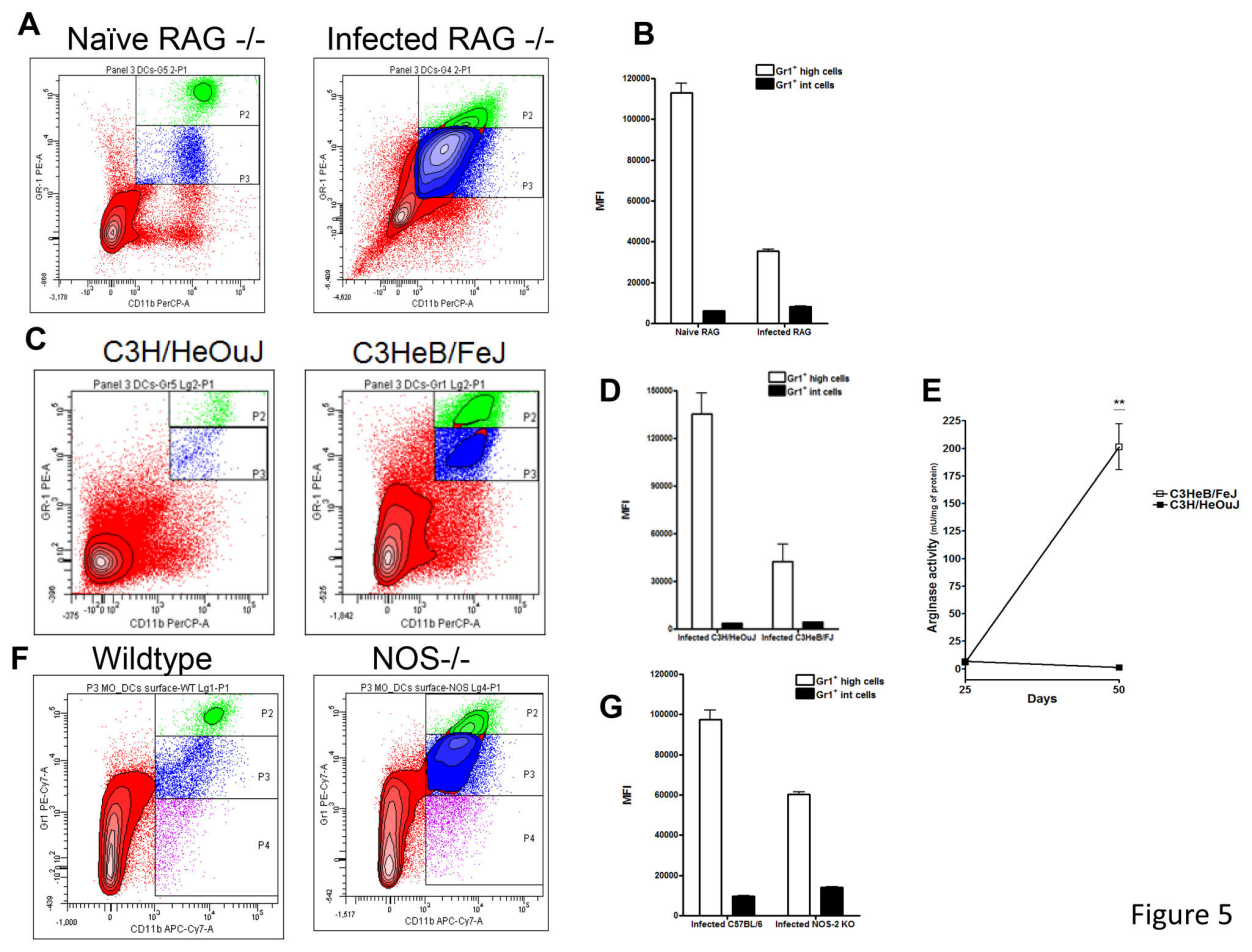
Significant numbers of Gr1^int^ CD11b^+^ cells were also found in RAG2-/- and C3HeB/FeJ mice. The influx of Gr1^int^CD11b^+^ cells was evaluated in immunocompromised RAG-/- (A), and immunocompetent C3HeB/FeJ mice (C) and NOS-/- (F). Again, large numbers of Gr1^int^CD11b^+^ cells were observed in these three mouse strains and significant differences were observed in the Gr1^+^ MFI (B, D, G). Similar to NOS2 -/- mice, arginase activity was also increased in C3HeB/FeJ undergoing lung necrosis (E). In contrast, C3H/HeOuJ did not have major arginase activity. Similar to NOS2 -/- mice, arginase activity was also increased in C3HeB/FeJ undergoing lung necrosis (E). Results are expressed as the mean values of mean fluorescence intensity (MFI) or arginase activity (± SEM, n=5) in the Lung. **Student t-test, p<0.001.

Given these observations, we posed the question as to whether these observations were unique to the NOS2 -/- model, or were a common feature in models in which lung necrosis occurs. To address this question, we used immunocompetent C3HeB/FeJ mice and immunodeficient Rag2 -/- mice as additional possible examples, given the knowledge these both develop severe lung necrosis [19,30-32]. As shown in Figure 5, similar results were obtained in these C3HeB/FeJ (Figure 5A, B), Rag2 -/- (Figure 5C, D) and NOS-/- mice (Figure 5F, G). In infected Rag2 -/- mice a very prominent Gr-1^int^CD11b^+^ population was by far the largest (Figure 5A, B). In C3HeB/FeJ mice, two clearly distinct Gr1^+^ CD11b^+^ populations of relatively equal size were only observed in the necrotic-prone C3HeB/FeJ mice but not in control C3H/HeOuJ devoid of necrosis (Figure 5C, D). Lastly, in NOS-/- mice compared to wild-type mice two clearly distinct Gr1^+^ CD11b^+^ populations were observed with the Gr-1^int^CD11b^+^ population being larger than the Gr-^hi^CD11b^+^ (Figure 5F, G) population.

### Enhanced arginase-1 activity is also present in the lungs of necrotic-prone C3HeB/Fe and NOS -/- mice

L-arginine is catabolized either by arginase or nitric oxide synthase [27]. The absence of nitric oxide synthase in NOS2 -/- mice could therefore explain the enhanced arginase activity shown in Figure 4 B. However, enhanced arginase activity could be a common event present in other necrotic models that have increased Gr1^int^ cells. Thus, arginase activity was also evaluated in C3HeB/FeJ. Importantly, at later time points, arginase activity was also upregulated in necrotic-prone C3HeB/FeJ but not in control C3H/HeOuJ (Figure 5 E). These results suggest that Gr1^+^ arginase I^+^ cells located in the region close to the fibrous capsule could be participating in regulation of inflammation. 

### Sorted Gr1 cells induce activation of Th17 cells and CD4 proliferation

**Figure 6:**
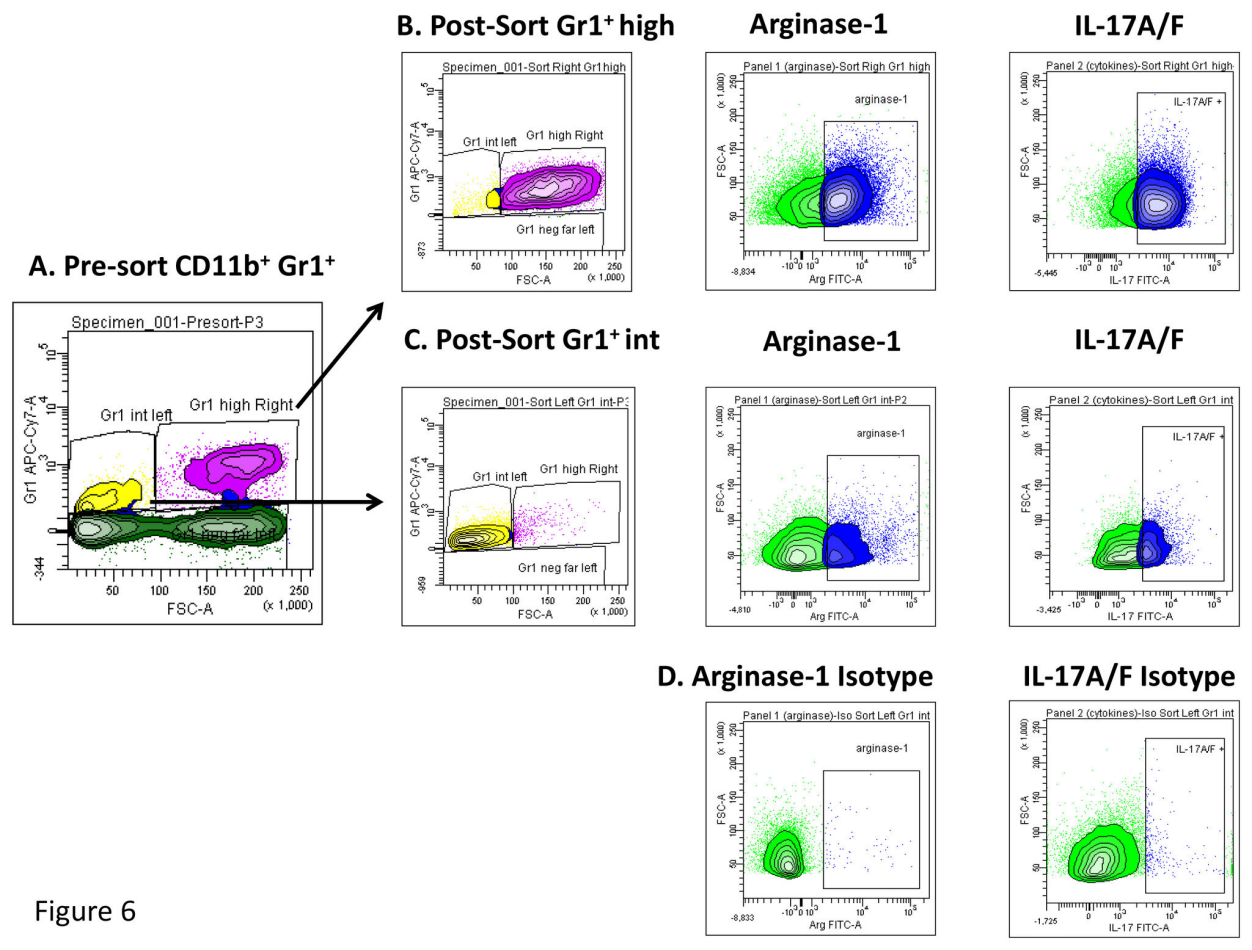
Gr1^int^ and Gr1^hi^ cells sorted from *M. tuberculosis* infected NOS-/- mice expressed arginase I and IL-17. Specific populations of Gr1^int^ or Gr1^hi^ cells were sorted from infected NOS-/- mice in an attempt to further characterize Gr1^int^ and Gr1^hi^ cell function during tuberculosis using flow cytometry. Panel A, demonstrates pre-sorted Gr1+CD11b+ cells which were then further characterized. Gr1^hi^ and Gr1^int^ sorted cells (B, C) both demonstrated high expression of arginase I and IL-17. Panel D shows the isotype controls for arginase-1 and IL-17A.

In an attempt to further characterize Gr1^int^ and Gr1^hi^ cell function during tuberculosis infection we conducted cell sorting of Gr1^int^ and Gr1^hi^ cells from naïve and infected NOS-/- mice. Figure 6 A, demonstrates pre-sorted Gr1+CD11b+ cells which were then further characterized. Gr1^hi^ and Gr1^int^ sorted cells (Figure 6 B, C) both demonstrated high expression of arginase-1 and IL-17. 

**Figure 7:**
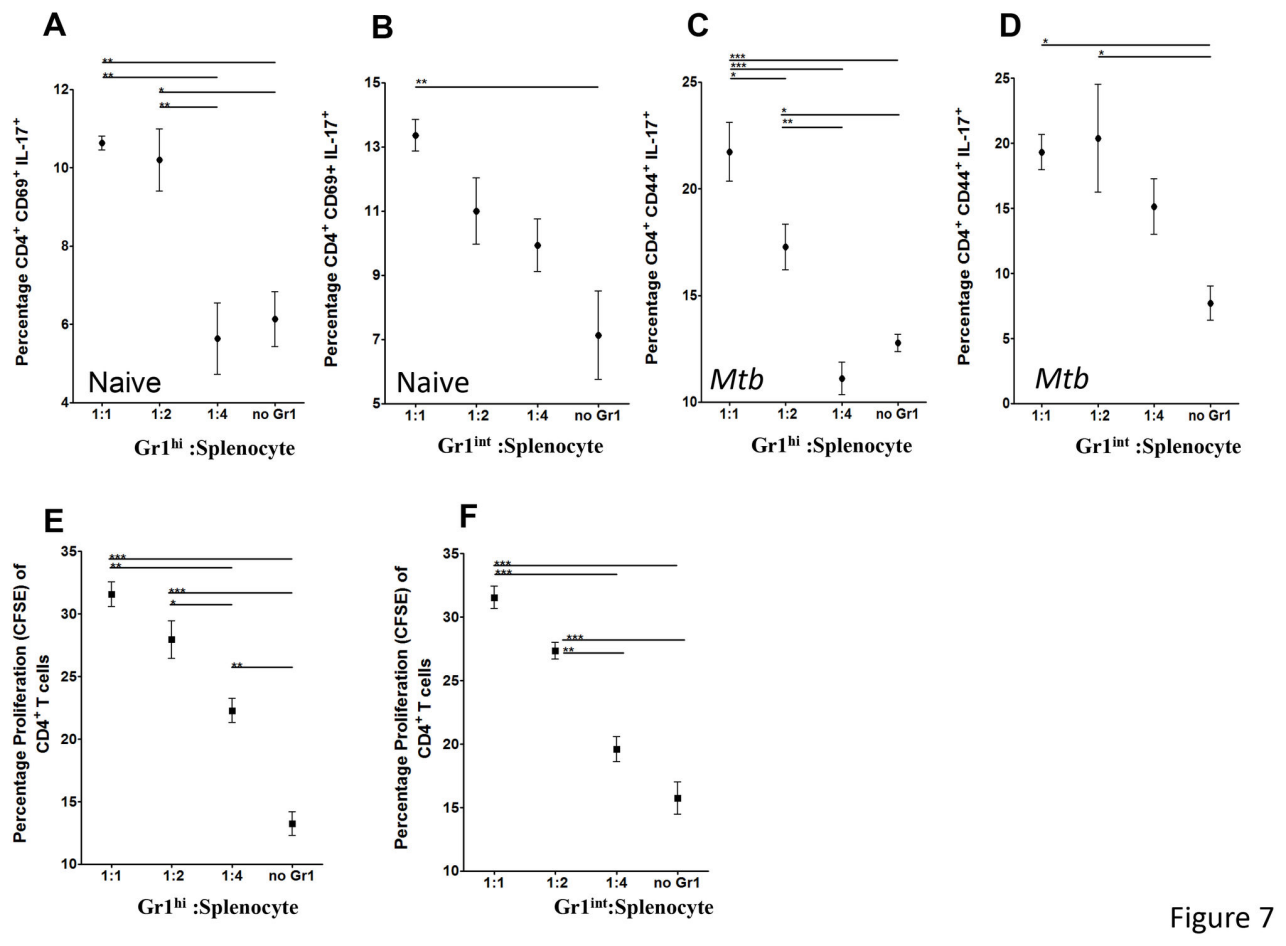
Gr1^int^ and Gr1^hi^ cells sorted from *M. tuberculosis* infected NOS-/- mice activate Th17 cells and induce proliferation of CD4^+^ T cells. Specific populations of Gr1^int^ or Gr1^hi^ cells were sorted from infected NOS-/- mice and placed into *in* *vitro* cultures with increasing numbers of either stimulated naïve or infected splenocytes. After 2 days of incubation T cells were evaluated for activation and memory profiles (A-D). Panel A -D shows lower ratios of Gr1^high^ (A and C) or Gr1^int^ (B and D) induce increased the percentage of activation of CD4^+^CD69^+^IL-17^+^ and memory CD4^+^CD44^+^IL-17^+^, when co-incubated with naïve (A and B) or *M. tuberculosis* infected splenocytes (C and D), respectively. Panel E and F shows Gr1 ^high^ and Gr1^int^ induced of proliferating CD4 T cells. However, as the splenocyte to Gr1^high^ or Gr1^int^ ratio increased the percentage of CD4+ proliferation was reduced. Results are expressed as the mean values of mean percentage of CD4^+^CD69^+^IL-17^+^, CD4^+^CD44^+^IL-17^+^ and proliferation. ANOVA and the Tukey post-test. *p<0.05, **p<0.01, ***p<0.001.

We then evaluated if sorted Gr1^+^ cells obtained from *M. tuberculosis* infected NOS2-/- mice, could modulate T cell activity as reported for MDSCs [26,33]. For this purpose, sorted Gr1^high^ and Gr1^int^ cells were separately co-incubated with either naïve splenocytes or splenocytes obtained from *M. tuberculosis* infected mice and thereafter evaluated for markers of activation CD69, memory CD44^hi^ and proliferation CFSE. After 2 days of culture, T cell cytokine production and surface activation markers were evaluated by flow cytometry. Surprisingly, both populations of sorted Gr1^+^ cells activated CD4^+^ but not CD8^+^ T cells (not shown), from naïve and infected animals (Figure 7A, B, C, D). In addition, co-incubation with Gr1^int^ cells led to a greater percent of CD4 T cells expressing the activation marker CD69 (Figure 7B, D). Interestingly, activated CD4 T cells produced the cytokine IL-17 A/F but not IFN-γ or IL-10 (data not shown), suggesting a polarization to Th17 type T cells. Co-incubation with Gr1^hi^ cells led to a greater percent of CD4 T cells expressing the activation marker CD69 and IL-17 at lower splenocyte to Gr1^hi^ ratios, however as the ratio of splenocytes increased CD4^+^CD69^+^IL-17 activation decreased (Figure 7A, C). 

Studies were then conducted to evaluate if the sorted Gr1^int^ or Gr1^hi^ cells from NOS-/- mice were able to induce proliferation. Figure 7 E and F demonstrates co-incubation with either population of sorted Gr1^+^ cells enhanced the proliferation of CD4 T cells (Figure 7E and F) at lower Gr1^+^ to splenocyte ratios but not CD8 T cells (not shown). Curiously, we noted as the ratio of Gr1^hi^ and Gr1^int^ to splenocyte increased reduced proliferation occurred (Figure 7E, F).

## Discussion

The results of this study provide further evidence that cells expressing intermediate levels of the Gr1 marker are a predominant subset of cells accumulating in the lungs of mice developing lung necrosis due to their inability to control infection with *M. tuberculosis*, whereas these cells appear to be in low numbers in similarly infected wild-type mice devoid of necrosis formation. In addition, we demonstrated this in NOS2 -/- mice and C3HeB/FeJ mice, but also in Rag2 -/- mice, the latter indicating that this response was independent of the need to generate acquired immunity. Furthermore, the presence of both populations of Gr1^+^ cells was associated with the expression of high levels of arginase-1, a key enzyme modulating the immune response by depleting arginine. 

We strongly suspect that neutrophils are the main cause of necrosis in both the mouse and guinea pig models of tuberculosis [3,5,34], and this is further emphasized by studies in humans [21,22]. To date we were assuming that our observations in NOS2 -/- and other murine models were due to the influx of neutrophils, based on simply measuring “Gr1-positive” cells, but clearly our current concept needs to be significantly modified based on the observations here that Gr1^hi^ and Gr1^int^ cells represent two distinct granulocyte subsets.

Whereas some of the Gr1^+^ cells are clearly neutrophils, an additional population of Gr1+ subsets, fall into the category of myeloid derived suppressor cells. These recognized as either a monocytic or granulocyte-like population that are implicated in the failure to control the growth of tumors [24], and also play a vital role in the control of chronic inflammatory diseases and autoimmunity. In the case of infectious diseases and specifically *Mycobacterium tuberculosis*, very little is known about these Gr1^int^ and Gr1^high^ cells and their role remains unknown. Certain disease states, such as those caused by *Pseudomonas* [35,36] seem to strongly induce MDSC, as does viral infection with HIV and SIV [24,37,38]. In the context of BCG vaccination, it was reported that MDSCs could be affecting vaccine efficacy through a nitric oxide dependent mechanism [39] and were unable to kill BCG or *M. smegmatis*. In addition, these studies showed they impaired T cell priming in the lymph node. Our results demonstrated that as the ratio of Gr1^high^ cells to splenocytes increased reduced proliferation was present. Furthermore in other chronic infections such as *Trypanosoma cruzi* and lupus prone MRL-Fas mice MDSC mediated suppression of CD4+ T cell proliferation [26,33,40]. However, in agreement with our observation in Wild-type C57BL/6 and C3H/HeOuJ, Gr1^int^ cells represented a minority of the total CD11b^+^ cells. In contrast, Gr1^int^ cells were increased only in tuberculosis murine models undergoing necrosis. 

 MDSCs appear to be able to modulate T cell functions [41,42], and may possibly trigger regulatory T cells [38], an increasingly important subset in tuberculosis [3,43,44], and this could be interpreted as a mechanism to limit inflammation in the lungs and hence reduce lung damage. This is far from clear however, and while a report [41] shows protection against sepsis by adoptive transfer of these cells, another shows that transfer into normal mice damages the lungs [36]. Mechanistically, the immunomodulatory activity present in MDSCs has been principally attributed to the expression of arginase and nitric oxide synthase [24,27]. Arginine depletion leads to decreased T cell expression of CD3ζ in tuberculosis and cancer patients [45-48], and can cause T cell growth arrest [49]. In the current tuberculosis murine models, arginase activity correlated with the development of lung necrosis and the presence of Gr1^int^ cells. In fact, arginase-1 was co-localized to the rim of necrotic granulomas where Gr1^+^ cells were also detected. In our studies in necrosis prone mice we identified large, vacuolated cells with a monocytic morphology, expressing arginase-1 and IL-17 cells identified by flow cytometry. However, further studies are required to determine the nature of the arginase-1 expressing cells. The presence of arginase-expressing cells surrounding necrotic areas has been previously reported for other models, including human tuberculosis [50,51]. It would be interesting to evaluate if arginase has a causal role in lung necrosis, by indirectly affecting endothelial function in blood vessels in close proximity to granulomas [52]. 

Even though most studies have reported that MDSCs suppress the immune response, particularly T cells, in some recent models it has emerged that MDSCs could actually activate and induce proliferation of T cells. Interestingly and in accordance with our results, in the experimental autoimmune encephalomyelitis (EAE) model it was reported that MDSCs activated Th17 cells. In turn, Th17 negatively affected the outcome of EAE by increasing pathology. Mechanistically, it was shown that Th17 induction in EAE was mediated by IL-1β. Furthermore, a complex relationship between TH17 cells producing IL-17 and MDSCs was recently unveiled in several cancer models. IL-17 was shown to increase the MDSCs-dependent induction of chronic inflammation by production of IL-1 in the tumor microenvironments. In our model, it could be envisioned that autocrine or paracrine IL-17 produced by Gr1^+^ or T cells, respectively could lead to inflammation and tissue damage, as well as increased bacterial burden. This uncontrolled tissue damage could also cause Gr1^int^ cells directly suppress other T cell subsets and to recruit regulatory T cells. However, this would need further validation with either IL-17 or IL-17R knockout mice or alternatively, with anti-IL-17 therapy. Our studies denoted as the splenocyte to Gr1^high^ or Gr1^int^ ratio increased the percentage of CD4+ proliferation reduced. These results suggest that Gr1^+^ subsets are either capable of suppression under these circumstances or alternatively the induced proliferation present at lower ratios was diluted out. Finally, it was recently reported that the origin of MDSCs could impact their immunomodulatory function. Whereas MDSCs obtained from a chronic peritonitis model activated T cells, peritoneal tumors induced MDSCs with suppressive functions. 

MDSC have been widely reported in human patients, mostly in the context of cancer, and in fact as new data accumulates there are probably subsets of MDSC themselves. The importance of neutrophils in the human immune response to tuberculosis is becoming more widely recognized, but what proportion of these cells is actually MDSC instead has not to our knowledge been investigated. Unfortunately, human neutrophils do not express Gr1, but other markers such as CD15, CD66b, CD115 and CD124 [53], could potentially allow distinction between true human neutrophils and MDSC. The role of Gr1^+^ cell subsets during *M. tuberculosis* infection merits further investigation.

## Materials and Methods

### Animals and infections

Six to 9 weeks old C57BL/6, NOS2 -/-, RAG2 -/-, C3HeB/FeJ and C3H/HeOuJ were purchased from the Jackson Laboratories (Bar Harbor, ME). This work was approved by the IACUC of Colorado State University. Animals were maintained in a BSL-3 facility at Colorado State University and had *ad libitum* access to water and chow. Mice were infected via aerosol with a low dose (approximately 100 CFUs) of *M. tuberculosis* H37Rv using a Glas-Col aerosol generator [27]. 

### Bacterial enumeration

At the indicated time points, mice were humanely euthanized via CO_2_ inhalation and organs harvested for CFU enumeration, histology, and flow cytometry analysis. Organs were homogenized in saline and serial dilutions were plated on 7H11 agar plates supplemented with OADC (BD Biosciences, San Jose, CA). After 3-4 weeks incubation at 37°C, CFUs were counted and the data expressed as the log_10_ numbers per target organ.

### Flow cytometry analysis

Cells were harvested from the lungs as described before [34]. Briefly, during necropsy, lungs were perfused with heparin (Sigma, St. Louis, MO). Thereafter, organs were minced and incubated for 30 min at 37°C with collagenase and DNAseI (Sigma), passed through a cell strainer and harvested by centrifugation. For antibody staining, cells were initially incubated with 10 µg/ml of FcBlock (anti-CD16/CD32, clone 93) for 20 min at 4°C. Thereafter, cells were stained with the following antibodies (all antibodies were from eBioscience, San Diego, CA) for 20 min at 4°C in the presence of FcBlock: anti-Gr1 (clone RB6-8C5), anti-CD11b (clone M1/70), anti-CD11c (clone N418), anti-CD14 (clone Sa2-8), anti-F4/80 (clone BM8), anti-Ly6-C (clone HK1.4), anti-CD4 (clone GK1.5), or anti-CD8 (clone 53-6.7). Data acquisition was performed on a LSR-II flow cytometer (BD) and 100,000 events were analyzed. Data was analyzed using FACSDiva version 6 (Becton Dickinson Instrumentation).

### Histology

Organs were perfused with 4% formaldehyde. Paraffin embedded tissues were stained with Hematoxylin and Eosin [27].

### Immunohistochemistry

Paraffin embedded slides were processed and after de-waxing with Histo-Clear (National Diagnostics, Atlanta, GA) and decreasing concentrations of ethanol, antigen retrieval was performed in a pressure cooker with DakoCytomation Target Retrieval Solution (DakoCytomation, Carpinteria, CA). Endogenous peroxidases and alkaline phosphatases were inactivated for 10 min with BLOXALL™ (Vector Laboratories, Burlingame, CA) and slides were blocked with 2.5% normal goat serum (Vector Laboratories) for 1 hr. Thereafter, slides were incubated O/N at 4°C with 1/40 rat anti-Gr1 (clone RB6-8C5, eBioscience) or rat IgG2b (isotype control, clone eB149/1OH5 eBioscience) diluted in 2.5% goat serum. After washing slides with TBS, they were incubated for 1 hr at RT with 1/1200 alkaline phosphatase-labeled, goat anti-rat IgG (Santa Cruz Biotechnology, Dallas, TX) diluted in 2.5% normal goat serum. The reaction was developed for 20 min with Vector® Red Alkaline Phosphatase Substrate (Vector Laboratories) and slides were counterstained with Hematoxylin QS (Vector Laboratories) for 30 sec. For arginase-1 expression, slides were processed similarly except for minor modifications. In this case, slides were blocked with ProteinBlock (DakoCytomation). The primary antibody was sheep anti-arginase-1 IgG (R&D, Minneapolis, MN), the isotype control was sheep IgG (R&D) and the secondary antibody was peroxidase-labeled donkey anti-sheep/goat IgG (AbD Serotec). Finally, the reaction was developed for 10 min with Liquid DAB substrate (X Biogenex, Freemont, CA). 

### Protein quantification

Lung lysates were centrifuged at 4°C for 10 min at 13,000 x g to remove debris and supernatants were quantified using the BCA assay (Pierce, Rockford, IL).

### Arginase activity

The level of arginase activity in lung lysates was performed using the QuantiChrom™ Arginase Assay Kit (BioAssays Systems, Hayward, CA). Briefly, protein concentration was normalized to100 µg/ml. As a control, an aliquot of normalized protein was heat-inactivated at 90°C for 10 min. Forty µl of untreated or heat-inactivated sample was incubated at 37°C for 60 min with 10 µl of 5X reagent. Thereafter, the reaction was processed as recommended by the manufacturer and analyzed in a plate reader (BioRad, Hercules, CA) at an absorbance of 430 nm. The absorbance of the heat-inactivated sample was subtracted from the respective untreated sample and then compared to urea standards. Activity is expressed as mU/mg of protein. 

### Cell sorting

To sort Gr1^int^ and Gr^high^, single cell suspensions were obtained from lungs as described above. After incubating with 5 µg/ml of Fc block (eBioscience) for 20 min at 4°C, cells were stained with PeCy7-labeled anti-CD11b (clone M1/70, eBioscience) and Alexa 700 labeled anti-Gr1 (clone RB6-8C5, eBioscience) as described above. Thereafter, cells were stained with 0.5 µg/ml 7-AAD (Invitrogen) for 5 min and sorted using a FACS Aria III (BD Biosciences) using the following strategy: doublets were gated out by FSC-A vs FSC-H and 7-AAD^+^ dead cells were excluded from the singlets population using FL3. Live singlets expressing CD11b+ were then analyzed based on FSC-A vs Gr1, in order to sort the two populations of Gr1^high^ and Gr1^int^. Sorted cells were analyzed for purity in a FACSAria. Additionally, cytokine production and arginase-1 expression was evaluated for both populations of freshly sorted Gr1^+^ cells, as follows: cells were incubated for 1 h at 37°C in a CO_2_ incubator with 2 µM monensin and 3 µg/ml brefeldin (eBioscience). After fixation and permeabilization using Fix/Perm and 1x permeabilization buffer, respectively (eBioscience), intracellular staining was performed with Alexa488-labeled anti-IL17A and F (clones TC11-18H10.1 and 9De.1C8, Biolegend) or FITC-labeled anti-arginase (R&D, same clone as for immunohistochemistyr), APC-labeled anti-IL-10 (clone JES5-16E3, eBioscience) or Alexa647-labeled anti-IL-12 (clone C17.8, eBioscience). As controls, cells were also labeled with the respective isotype control.

### Biological assays with sorted Gr1 populations

Cytokines and activation markers: T cells were obtained from naïve or *M. tuberculosis* infected mice. Thereafter, naïve T cells were stimulated with 0.5 and 5 µg/ml of anti-CD3 and anti-CD28, respectively. Gr1^high^ or Gr1^int^ cells were separately co-cultured at 37°C in a CO_2_ incubator with increasing ratios of splenocytes for 48 h. Cells were then incubated for 1 h at 37°C in a CO_2_ incubator with 2 µM monensin and 3 µg/ml brefeldin (eBioscience). Surface staining was performed with Alexa700-labeled anti-CD4 (clone RM 4-5) or Alexa700-labeled anti-CD8 (clone 53-6.7, eBioscience), Alexa 405-labeled anti-CD44 (clone IM7, eBioscience), PerCP-labeled anti-CD69 (clone H1.2F3, eBioscience) and APC-labeled anti-CD62L (clone MEL-14, eBioscience) followed by fixation and permeabilization as described above. In addition to the previously mentioned intracellular cytokines, cells were also stained with PE-Cy7 labeled-anti-IFN-γ (clone XMG1.2, eBioscience). Once again, respective isotype controls were used to determine non-specific binding. 

### Proliferation assay

T cells were obtained as described above and stained with CFSE (Invitrogen), following their protocol. Briefly, T cells were stained with 10 µM CFSE for 10 min at 37°C and after quenching the reaction with 3 volumes of medium, cells were washed 3 times with PBS. Stained T cells were incubated with increasing ratios of T cells per Gr1^high^ or Gr1^int^ cell for 7 days at 37°C in a CO_2_ incubator. Cells were then surfaced stained with PerCP-labeled anti-CD4 (same clone as above), Pe-Cy7-labeled anti-CD8 (same clone as above) and APC-labeled anti-CD3 e (clone 145-2C11, eBioscience) and analyzed using an LSR-II after gating on CD3^+^ CD4^+^ or CD3^+^CD8^+^.Proliferation was evaluated as a decrease in FL1 fluorescence over the basal conditions (in the absence of Gr1^+^ cells).

### Statistical analysis

Data are presented using the mean values from 5 mice per group and from values from replicate samples and duplicate or triplicate assays. Two-tailed, unpaired Student t-test or ANOVA was performed using GraphPad Prism 4 (GraphPad software, San Diego, CA). Significance was considered below p<0.05.
